# Psychosocial Well-Being of Informal Caregivers of Adults Receiving Home Mechanical Ventilation: A Scoping Review

**DOI:** 10.3390/jcm14176294

**Published:** 2025-09-05

**Authors:** Jakub Cichoń, Monika Homa, Lucyna Płaszewska-Żywko, Maria Kózka

**Affiliations:** 1 Department of Specialized Nursing, Faculty of Health Sciences, Jagiellonian University Medical College, 31-501 Kraków, Poland; 2 Aestimo s.c., 31-062 Kraków, Poland

**Keywords:** home mechanical ventilation, caregivers, well-being, home care, chronic respiratory failure

## Abstract

**Background/Objectives**: Home mechanical ventilation (HMV) is a therapeutic approach that enables individuals with chronic respiratory failure to be cared for in home settings, thereby improving their quality of life. However, it also imposes a substantial burden on informal caregivers. This scoping review aimed to explore and synthesize current research on the psychosocial well-being of informal caregivers of adults receiving HMV and to identify existing knowledge gaps. **Methods**: Following PRISMA-ScR guidelines, six electronic databases were systematically searched without language or date restrictions. Eligible studies were original, peer-reviewed publications focusing on informal caregivers of adults receiving HMV. Relevant data were extracted and analyzed. **Results**: A total of 38 studies met the inclusion criteria. The majority of caregivers were women, most commonly spouses or partners. Caregivers frequently experienced high levels of burden, anxiety, depression, fatigue, and reduced quality of life. Common challenges included social isolation, sleep disturbances, and financial difficulties. Caregivers employed a range of coping strategies, both adaptive and maladaptive. Many reported unmet needs, particularly in the areas of psychological, informational, and professional support. **Conclusions**: Providing care for individuals receiving HMV is complex and demanding. While some caregivers find meaning and fulfillment in their role, many experience significant physical, emotional, and psychological challenges. These findings highlight the urgent need for comprehensive, individualized interventions aimed at reducing caregiver burden, enhancing quality of life, and ensuring better integration of caregivers into the broader care continuum.

## 1. Introduction

Home mechanical ventilation (HMV) represents a significant form of respiratory support for individuals with chronic respiratory failure. This approach facilitates the continuation of therapy outside the hospital setting and serves as an alternative to institutional care. HMV is particularly beneficial for individuals with respiratory and neuromuscular disorders, such as amyotrophic lateral sclerosis, muscular dystrophies, as well as chronic obstructive pulmonary disease, as it enables them to remain in a familiar and supportive home environment, surrounded by family and close friends [1,2]. It has been shown to improve quality of life of ventilator-assisted individuals (VAIs), enhance independence, reduce the risk of hospitalization, and extend survival [3,4,5]. Furthermore, patients generally report high levels of satisfaction with receiving care at home [6].

HMV can be provided either invasively (IV) or non-invasively (NIV). IV is delivered via tracheostomy tube, whereas NIV uses a range of interfaces, most commonly oronasal and nasal masks. HMV works by supporting or fully taking over the patient’s ventilation, ensuring sufficient oxygenation and removal of carbon dioxide [2].

Despite the numerous benefits of HMV for VAIs, home-based care significantly affects the functioning of the entire family, particularly those directly involved in the caregiving process. Providing long-term, daily care for VAIs living at home poses substantial challenges for informal caregivers. The transition from hospital to home care requires a reorganization of the family’s daily life, acquisition of technical skills related to equipment use (including operating ventilators, responding to alarms, and managing a tracheostomy), preparedness for emergency situations, and assuming responsibility for the holistic, continuous care of a loved one [5].

Caregivers are exposed to both physical and psychological demands. A high level of responsibility, persistent stress, constant readiness to act, and daily caregiving tasks have been shown to contribute to a decline in caregivers’ quality of life and an increased risk of anxiety and depression [7].

Although access to HMV is expanding in many countries [8] and interest in this area of research is growing, knowledge about the experiences and needs of informal caregivers remains limited. There is an insufficient number of comprehensive studies addressing the psychological, social, and physical dimensions of caregiving for VAIs living at home.

### Aim and Research Questions

The aim of this scoping review was to explore and map the nature, extent, and scope of existing research on the psychosocial well-being of informal caregivers of adults receiving HMV. Furthermore, the review sought to provide a comprehensive overview of the current state of knowledge and to identify gaps in the literature that could inform future research in this field.

The study was guided by the following research questions:

(a) Which aspects of the psychosocial well-being of informal caregivers of adults receiving HMV are most frequently explored in the literature?
(b) What research methods and instruments are employed to investigate the psychosocial well-being of these caregivers?
(c) What knowledge gaps can be identified based on the literature?

## 2. Materials and Methods

This review was conducted and reported in accordance with the PRISMA extension for scoping reviews (PRISMA-ScR) guidelines [9]. A study protocol was developed in advance to guide the review process and ensure alignment in every process step. The protocol was not registered; however, it is available in “Supplementary File S1”.

Initially, the research questions were formulated broadly to capture the most relevant aspects of the psychosocial well-being of informal caregivers of individuals receiving HMV, thereby informing both the search strategy and the overall scope of the analysis.

### 2.1. Search Strategy

The search strategy was developed collaboratively by the authors in consultation with an academic librarian. This process involved the identification and selection of relevant keywords, which were subsequently used to construct search strings employing the Boolean operator “OR”. Two primary conceptual domains were defined: terms related to “caregivers” and those related to “mechanical ventilation”. These two sets of terms were then combined using the Boolean operator “AND” to narrow the results and retrieve publications addressing both topics concurrently. The complete search strategy is provided in “Supplementary File S2”.

A comprehensive literature search was conducted across the following databases: APA PsycInfo (via EBSCO), Embase, MEDLINE (via PubMed), Scopus, CINAHL Ultimate, and Web of Science. To maximize the search scope, no filters for language or publication date were applied during the database searches. The search results are current as of 22 November 2024.

### 2.2. Study Selection and Eligibility Criteria

Zotero software for macOS (version 7.0.13; Corporation for Digital Scholarship, Vienna, VA, USA) was used for reference management. Duplicate records were removed, and titles and abstracts were screened for eligibility according to predefined inclusion and exclusion criteria by the first and second authors (J.C. and M.H.). In cases of disagreement regarding study inclusion, the articles were discussed with a senior academic (L.P.-Ż.), and final decisions were made by the last author (M.K.). Discrepancies were infrequent, and inter-rater agreement was not computed, as it is not a standard requirement for scoping reviews.

Studies were deemed eligible for full-text review if the following inclusion criteria were met:

(1) Primary, peer-reviewed, and original full-length research articles published in a scientific journal;
(2) Studies that report evidence on the psychosocial well-being of informal, unpaid caregivers for adults receiving HMV;
(3) Studies that employed quantitative, qualitative, or mixed-methods research methods;
(4) Full-text available online.

Studies were excluded if they met at least one of the following criteria:

(1) Secondary research (e.g., systematic reviews, scoping reviews, and narrative reviews), nonoriginal publications (e.g., editorials, commentaries, and letters to the editor), gray literature (e.g., dissertations, theses, study protocols, conference abstracts, and book chapters), or single case studies;
(2) Studies involving heterogeneous populations, in which data specific to informal caregivers of adults receiving HMV were not separately reported or extractable;
(3) Studies on caregivers of individuals using continuous positive airway pressure (CPAP) or home oxygen therapy only;
(4) Published in a language other than English.

For studies in which the titles and abstracts did not contain sufficient information, a full-text review was performed.

### 2.3. Charting Process and Reporting of Results

The data extraction sheet was developed by the first author (J.C.) in collaboration with the co-authors. Extracted data included the following:

(1) Publication details;
(2) Methods;
(3) Characteristics of the study population;
(4) Key concepts and findings.

Data analysis was conducted in two stages. First, tabular summaries were created to provide an overview of the included studies. Subsequently, in accordance with current methodological guidance [9,10], a qualitative content analysis was performed using an inductive coding approach. The first and second authors (J.C. and M.H.) independently coded the extracted data. Any disagreements were resolved through discussion with senior authors (L.P.-Ż. and M.K.). Inter-coder reliability was not formally computed. The extracted data were organized into key concepts and study characteristics relevant to the review’s objectives. Example codes included caregiver burden, anxiety, depression, stress, coping, support, and quality of life. This descriptive approach ensured methodological transparency and was consistent with the exploratory nature of the scoping review.

## 3. Results

### 3.1. Study Characteristics

**Figure 1:**
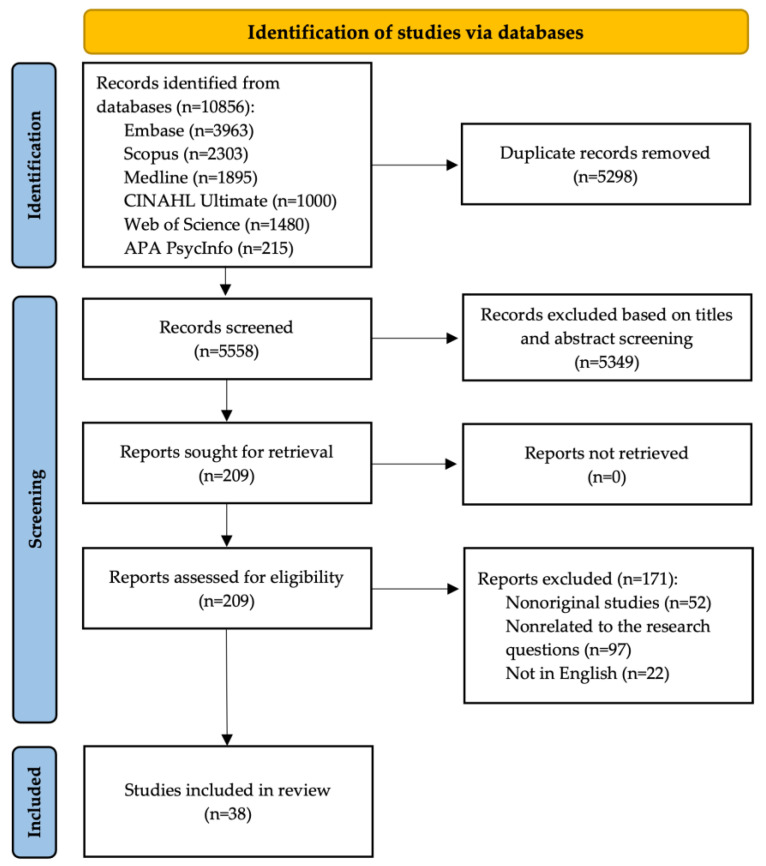
Flow diagram (adapted from [12]).

In total, 10,856 records were identified, of which 5558 titles and abstracts were screened, 209 full-text articles were assessed for eligibility, and 38 studies met the inclusion criteria. Details of the study selection process are presented in Figure 1.

The studies included in this review were published between 1991 and 2024 and comprised quantitative (*n* = 17), qualitative (*n* = 13), and mixed-method (*n* = 8) designs. The majority were conducted in the United States (*n* = 5), Iran (*n* = 4), Germany (*n* = 3), Italy (*n* = 3), Taiwan (*n* = 3), Turkey (*n* = 3), and the United Kingdom (*n* = 3), as well as part of a multicountry study [11]. For seven studies, the study period coincided with the COVID-19 pandemic, which may have influenced the study outcomes. All included studies reported ethics approval where applicable.

### 3.2. Sociodemographic Findings

Sociodemographic data were at least partially available in all studies. The number of informal caregivers studied ranged from 5 to 250. In studies where sex was reported, the majority of caregivers were women (60% to 100%), and the mean age of the caregivers ranged from 38.7 to 62 years. The most often caregivers were spouses or partners (*n* = 16), followed by parents (*n* = 8) and adult children (*n* = 4) of VAIs.

VAIs living at home use both IV and NIV (here referring specifically to bilevel positive pressure ventilation), with the proportions varying between studies. The reported prevalence of IV ventilation ranged from 12% to 100%, and NIV ventilation ranged from 10% to 100%. Some of the studies included only individuals with either IV or NIV. Men comprised 45% to 100% of VAIs, but only three studies (including one control group) included men as a minority. The mean age of the VAIs ranged from 23.20 to 69.1 years.

Table 1 presents demographic data on informal caregivers and VAIs, as well as details concerning mechanical ventilation. The key concepts and measures of the included studies are summarized in Table 2.

### 3.3. Caregiver Burden

Caregivers’ burden is among the most extensively studied areas in caregiving research. In quantitative studies to evaluate burden, standardized tools such as the Zarit Burden Interview (ZBI, *n* = 7), Caregiver Strain Index (CSI, *n* = 4), Caregiver Burden Inventory (CBI, *n* = 2), Burden Scale for Family Caregivers (BSFC, *n* = 2), and Caregiver Burden Scale (CBS, *n* = 2) were used most often. Sample mean scores ranged from 38.5 ± 16.3 to 71.30 ± 4.12 on the ZBI (possible range 0–88) [13,14] and from 13.20 ± 10.18 to 25.38 ± 10.47 on the BSFC (possible range 0–84) [15,16].

Numerous quantitative studies have indicated a significant level of caregiver burden [14,17]. Some studies have identified gender differences in caregiver burden, with women reporting higher levels of burden compared to men [7,13,18].

Findings on caregiver burden in relation to the type of ventilation (IV vs. NIV) were inconclusive: while some studies reported significant differences [7,19], others found no such association [13,20].

Qualitative and mixed-method studies deepen the understanding of the multidimensional nature of the burden, pointing to aspects such as limitations in social life, isolation, lack of freedom [1,5,15,21,22,23,24], lack of free time [21,24], sleep disorders [21,23,24,25,26], and financial burden and employment issues [1,15,21,22,24,27,28,29,30,31,32] as major factors affecting caregivers’ experiences. Changes in the level of caregiver burden over time vary, highlighting its complexity and the influence of multiple contributing factors [30,33].

### 3.4. Mental Health and Quality of Life

Mental health problems, including anxiety, depression [11,13,22,23,34,35], fatigue [11,21,23,26,36], guilt and self-blame [1,21,23], as well as stress [1,25,29,31], are frequently reported by caregivers. Negative impacts on physical health have also been reported among long-term caregivers [24].

A variety of instruments have been employed to assess quality of life, including the World Health Organization Quality of Life Scale—Short Form (WHOQOL-BREF), 36-Item Short Form Health Survey (SF-36), EQ-5D, and the Munich Quality of Life Dimensions List. Several studies have reported a reduction in caregivers’ quality of life compared to that of the general population. For example, reported mean quality of life scores were 70.27 ± 14.95 on the SF-36 (possible range 0–100) [20], 70.4 ± 22.8 on the EQ-5D (possible range 0–100) [13], and 46.65 ± 8.52 on the WHOQOL-BREF (possible range 0–100) [19]. Furthermore, a negative correlation has been observed between quality of life and the burden of caregiving [13,19,20,26].

**Table 1:** Characteristics of caregivers and ventilator-assisted individuals living at home.

Publication		Caregivers of Individuals Receiving HMV				VAIs Living at Home			
Authors	Year	*n*	Age (y)	Female (%)	Relationship to Patient (%)	Age (y)	Male (%)	Type of Ventilation (%)	Duration of HMV
*Quantitative studies:*									
Thomas et al. [37]	1992	44	47.3 ± 13.5	61%	27% mothers, 18% husbands, 14% wives, 14% fathers, 14% daughters, 5% sisters, and 9% others	43 ± 22.8	59%	n/a	20.5 (IQR n/a) mo
Ferrario et al. [18]	2001	40	56.50 ± 14.30	63%	55% spouses and 45% others	65 ± 9.1	73%	n/a	24.3 ± 20.1 mo
Kaub-Wittemer et al. [27]	2003	52	n/a	81%	98% spouses and 2% daughters	60.0 ± n/a (NIV); 61.6 ± n/a (IV)	79%	60% NIV; 40% IV	13.8 ± n/a mo (NIV); 34.6 ± n/a mo (IV)
Tsara et al. [28]	2006	50	47.98 ± 14.2	majority were female	42% spouses, 39% children, and 19% others	61 (IQR n/a)	predominantly male	88% NIV; 12% IV	3.5 ± 2.4 y
Kim & Kim [20]	2014	83	n/a	n/a	n/a	59.66 ± 10.97	63%	77% IV; 23% NIV	25.56 ± 19.91
Liu et al. [38]	2017	80	50.59 ± 14.92	73%	45% children, 33% spouses, 17% sons/daughters, and 5% others	63.75 ± 16.95	61%	100% IV	32.04 ± 33.43 mo
Jacobs et al. [39]	2021	34	59.5 ± 15.9	65%	38% parents, 29% partners, 24% children, and 9% siblings	53.8 ± 21.3	56%	100% IV	51.2 (IQR 28–199) mo
Liang et al. [40]	2022	n/a	n/a	n/a	n/a	61.94 ± 19.50	53%	n/a	n/a
Pandian et al. [11]	2022	34	43.3 ± 15.9	n/a	n/a	51.3 ± 12.2	n/a	n/a	n/a
Volpato et al. [33]	2022	66	n/a	n/a	n/a	69.1 ± 8.6	45%	100% NIV	n/a
Esmaeili et al. [17]	2023	51	46.60 ± 12.24 (Inter.); 43.56 ± 9.83 (Ctrl.)	n/a	n/a	54.85 ± 15.24 (Inter.); 52.78 ± 13.89 (Ctrl.)	50% (Inter.); 48% (Ctrl.)	100% IV	n/a
Karagün et al. [19]	2023	250	n/a	n/a	n/a	n/a	n/a	34% NIV	n/a
Marcus et al. [41]	2023	34	59.5 ± 15.9	65%	38% parents, 29% partners, 24% children, and 9% siblings	53.8 ± 21.3	n/a	100% IV	51.2 (IQR 28–199) mo
Tülek et al. [13]	2023	66	n/a	n/a	n/a	n/a	n/a	53% IV; 10% NIV	n/a
Kavand & Asgari [14]	2024	51	46.60 ± 12.24 (Inter.); 43.56 ± 98.30 (Ctrl.)	78%	45% spouses, 35% children, and 20% parents	54.85 ± 15.24 (Inter.); 52.78 ± 13.89 (Ctrl.)	51%	100% IV	n/a
Lee et al. [34]	2024	59	62 (IQR 55–70)	80%	56% spouses, 39% children, and 5% other family members	62 (IQR 55–70)	59%	95% IV	n/a
Płaszewska et al. [7]	2024	58	53.81 ± 13.73	66%	n/a	56.47 ± 14.62	52%	64% IV; 36% NIV	3.54 ± 2.63 y
*Qualitative studies:*									
Findeis et al. [21]	1994	13	50.92 ± 14.15	n/a	31% wives, 23% husbands, 15% mothers, 15% parents, 8% fathers, and 8% girlfriends	42 ± 18.24	58%	n/a	n/a
van Kesteren et al. [1]	2001	43	n/a	n/a	n/a	36.74 ± 15.88	63%	68% IV; 32% NIV	83.89 ± 40.57 mo
Akiyama et al. [35]	2006	12	56.1 ± 13.2	83%	75% spouses, 17% mothers, and 8% daughters	n/a	n/a	83% IV; 17% NIV	n/a
Sundling et al. [42]	2009	8	range 40–74	75%	100% spouses	range 45–75	71%	100% NIV	range 3–15 mo
Huang & Peng [29]	2010	15	57.7 ± n/a	60%	33% children, 27% spouses, 27% mothers, and 13% daughters-in-law	n/a	n/a	n/a	n/a
Dale et al. [5]	2018	14	n/a	100%	74% spouses	54.89 ± 18.21		53% IV; 47% NIV	n/a
Dickson et al. [43]	2018	8	51.13 ± 8.68	88%	38% spouses, 38% siblings, and 24% mothers	44.25 ± 15.94	88%	n/a	range 4–20 y
Schaepe & Ewers [16]	2018	15	62 ± 11.75	80%	60% spouses, 20% mothers, 13% children, and 7% sisters	n/a	n/a	87% IV; 13% NIV	11.15 ± 13.46
MacLaren et al. [36]	2019	6	n/a	n/a	83% partners and 17% parents	44 (IQR n/a)	93%	79% NIV; 21% IV	21% <1 y, 43% 1–9 y, and 36% >10 y
Yamaguchi et al. [30]	2019	14	53.86 ± 4.31	86%	86% mothers and 14% fathers	23.20 ± 4.97	100%	60% NIV; 40% IV	n/a
Esmaeili et al. [22]	2022	9	39.5 ± 6.64	n/a	67% children, 22% spouses, and 11% parents	n/a	n/a	100% IV	n/a
Khankeh et al. [31]	2022	12	n/a	n/a	25% parents, 25% children, 17% siblings, 17% spouses, and 16% others	n/a	n/a	n/a	n/a
Aydin et al. [23]	2024	21	38.7 ± 10.3	81%	52% mothers, 24% daughters, 9% fathers, 5% grandmothers, 5% siblings, and 5% sons	n/a	48%	100% IV	n/a
*Mixed-method studies:*									
Smith et al. [44]	1991	20	51.25 ± 14.41	n/a	35% wives, 20% husbands, 20% mothers, 5% fathers, 5% sons, 5% daughters, 5% brothers, and 5% VAIs described themselves as a caregiver	49.10 ± 17.76	75%	50% IV; 50% NIV	42.80 ± 67.38 mo
Moss et al. [45]	1993	19	n/a	90%	n/a	57 ± n/a range 36–78	79%	84% IV; 16% NIV	20 ± n/a mo range 3–70 mo
Smith et al. [32]	1994	20	20–74	65%	50% spouses, 25% parents, 15% children, 5% close relatives, and 5% described themselves as a caregiver	18–74	n/a	55% IV; 45% NIV	45% ≤1 y, 35% 2–4 y, 15% 5–9 y, and 5% 26 y
Marchese et al. [25]	2008	77	n/a	81%	71% spouses, 23% parents, 4% sons, and 2% close friend	58.2 ± 17.5	70%	100% IV	n/a
Evans et al. [24]	2012	21	53.86 ± 14.30	62%	24% mothers, 24% fathers, 24% wives, 9% daughters, 9% sons, 5% husbands, and 5% sisters	45 ± 13	n/a	100% IV	8 ± 5
Baxter et al. [26]	2013	16	n/a	n/a	69% wives, 19% husbands, 6% daughters, and 6% other family members	n/a	n/a	100% NIV	n/a
Klingshirn et al. [15]	2022	5	52.8 ± 5.36	80%	60% parents and 40% spouses	46.86 ± 15.40	64%	71% IV; 29% NIV	11.67 ± 8.0
Sheers et al. [46]	2024	12	n/a	n/a	n/a	n/a	78% (Inter.); 82% (Ctrl.)	100% NIV	n/a

Abbreviations: *n*—sample size; IV—invasive ventilation; NIV—non-invasive ventilation; y—years; mo—months; IQR—interquartile range; Inter.—intervention group; Ctrl.—control group; HMV—home mechanical ventilation; VAIs—ventilator-assisted individuals; n/a—not applicable. Note: Data are presented as the means ± standard deviations, medians (IQRs), ranges, or frequencies, as appropriate. All percentage values were rounded to the nearest whole number. Only data related to caregivers of individuals receiving HMV and VAIs living at home were included, even though other groups were described in the original studies.

**Table 2:** Key concepts and measures of the included studies.

Authors	Year	Country	Study Period	Instruments Used to Assess Caregivers	Key Concepts Related to Caregivers of VAIs Living at Home
*Quantitative studies:*					
Thomas et al. [37]	1992	United States	1989	Caregiver Needs	Caregiver needs
Ferrario et al. [18]	2001	Italy	n/a	Family Strain Questionnaire	Family strain
Kaub-Wittemer et al. [27]	2003	Germany	n/a	Author-developed questionnaire; Profile of Mood States; Munich Quality of Life Dimensions List	Quality of life; depression; fatigue; vigor; anger; patient care; home and personal situation; partnership; burden
Tsara et al. [28]	2006	Greece	n/a	Family Burden Questionnaire	Burden; coping
Kim & Kim [20]	2014	South Korea	August 2008–April 2009	Zarit Burden Interview; 36-Item Short Form Health Survey	Burden; quality of Life
Liu et al. [38]	2017	Taiwan	June–December 2010	Burden Assessment Scale	Burden
Jacobs et al. [39]	2021	Israel	May 2016–April 2018	Caregiver Strain Index	Strain; cost of care
Liang et al. [40]	2022	Taiwan	November 2016–June 2017	Family Caregiver Belief Scale	Beliefs
Pandian et al. [11]	2022	Cross-country	n/a	Author-developed questionnaire	Anxiety; fatigue; mood; loneliness
Volpato et al. [33]	2022	Italy	May 2015–December 2017	Caregiver Burden Inventory; Caregiver Burden Scale; Zarit Burden Interview	Home adaptation vs. outpatient adaptation; burden; satisfaction
Esmaeili et al. [17]	2023	Iran	June 2020–January 2022	Zarit Burden Interview	Effect of training; burden
Karagün et al. [19]	2023	Turkey	September 2019–April 2020	Hospital Anxiety and Depression Scale; World Health Organization Quality of Life Scale—Short Form; Zarit Burden Interview	Anxiety; depression; quality of life; burden
Marcus et al. [41]	2023	Israel	May 2016–April 2018	Caregiver Strain Index	Strain
Tülek et al. [13]	2023	Turkey	September 2015–March 2016	Hospital Anxiety and Depression Scale; EQ-5D; Zarit Burden Interview; Multidimensional Scale of Perceived Social Support	Burden; quality of life; anxiety; depression; social support
Kavand & Asgari [14]	2024	Iran	July 2020–November 2021	Functional skills checklist; Zarit Burden Interview	Effect of training; burden
Lee et al. [34]	2024	South Korea	August–October 2022	Patient Health Questionnaire; Preparedness for Caregiving Scale; Caregiver Competence Scale	Depression; emotional difficulties; care preparedness; care capability
Płaszewska et al. [7]	2024	Poland	n/a	Caregiver Burden Scale; Social Support Scale; Brief COPE	Burden; social support; coping
*Qualitative studies:*					
Findeis et al. [21]	1994	United States	n/a	Semi-structured interviews; List of caregiving tasks; Caregiving Appraisal Scale	Caregiving tasks; burden; impact of caregiving; mastery of the caregiving role; satisfaction
van Kesteren et al. [1]	2001	Netherlands	January 1996–May 1998	Semi-structured interviews	Strain; receiving information; unexpected problems; obstacles; expected help; change in life; choosing respiratory support again
Akiyama et al. [35]	2006	Japan	August 2001–September 2002	Semi-structured interviews	Hesitation and regret; support
Sundling et al. [42]	2009	Sweden	2002–2005	In-depth interviews	Getting to know the ventilator; embracing the ventilator; being on the ventilator on a 20–24 h basis
Huang & Peng [29]	2010	Taiwan	January–December 2007	In-depth interviews	Adaptation
Dale et al. [5]	2018	Canada	n/a	Semi-structured interviews	Facilitators and barriers
Dickson et al. [43]	2018	United Kingdom	n/a	Semi-structured interviews	Negotiating boundaries of care and finding a “fit”
Schaepe & Ewers [16]	2018	Germany	June 2014–June 2015	Semi-structure interviews; Burden Scale for Family Caregivers	Burden; contribution of family caregivers to safety in HMV
MacLaren et al. [36]	2019	United Kingdom	2015–2016	Semi-structured interviews	Care; personal impact of caring.
Yamaguchi et al. [30]	2019	Japan	March 2013–September 2016	Serial interviews	Family relationships
Esmaeili et al. [22]	2022	Iran	November 2019–May 2020	Semi-structured interviews	Educational, psychological, and economical needs
Khankeh et al. [31]	2022	Iran	2015, 2019	Semi-structured interviews	Challenging care with stress and ambivalence; step-by-step care delegation
Aydin et al. [23]	2024	Turkey	April 2019–June 2019	Semi-structured interviews	Physiology; self-concept; role–function; interdependence
*Mixed-method studies:*					
Smith et al. [44]	1991	United States	n/a	Semi-structured interviews; Family Coping Scale; Family Apgar	Adaptation; coping; perceptions of family function
Moss et al. [45]	1993	United States	n/a	Structured interviews	Decision on HMV; benefits and burdens; costs of HMV; attitudes toward HMV
Smith et al. [32]	1994	United States	n/a	Semi-structured interviews; Learning Needs Checklist; Caregiver Reaction Inventory; Family Coping Strategies Scales; Family Apgar	Responsibilities; learning needs; reactions to caring and caregiving; coping; perceptions of family function
Marchese et al. [25]	2008	Italy	January 1995–December 2004	Structured interviews; Caregiver Strain Index	Benefits and burdens; attitudes
Evans et al. [24]	2012	Canada	n/a	Semi-structured interviews; Caregiver Burden Inventory	Sense of duty; restriction on day-to-day life; burden; training and education; paid support
Baxter et al. [26]	2013	United Kingdom	n/a	Semi-structured interviews; 36-Item Short Form Health Survey; Caregiver Strain Index	Quality of life; strain; impact of NIV; burden; role change; difficulty having time away; professional support
Klingshirn et al. [15]	2022	Germany	June 2019–August 2020	Semi-structured interviews; Burden Scale for Family Caregivers	Burden; daily care; social relationships and participation; safety; care coordination; improvement
Sheers et al. [46]	2024	Australia	August 2020–August 2021	Semi-structured interviews; Zarit Burden Interview	In-home model of NIV initiation vs. single-day admission; advantages and disadvantages of care; burden and barriers; benefits and enablers

Abbreviations: HMV—home mechanical ventilation; NIV—non-invasive ventilation; VAIs—ventilator-assisted individuals; n/a—not applicable. Note: Sociodemographic data questionnaires were not included in the table. Only data related to caregivers of individuals receiving HMV and VAIs living at home were included, even though other groups were described in the original studies.

### 3.5. Coping and Spirituality

Caregivers use a variety of coping strategies, both positive (reorientation of goals, planning, acceptance, and active coping) and negative (resignation) [7,28,32]. A considerable number of caregivers report satisfaction with their caregiving role and value the opportunity for loved ones to remain in the comfort of their own homes [1,29,30,45]. Some caregivers engaged in spiritual practices as a coping mechanism [18,23,29].

### 3.6. Caregivers’ Needs and Support Expectations

A review of the literature identified caregivers’ needs related to support from family members and friends [5,13,18,21,28,29,30], informational and educational support [14,21,22,23,29,37,46], continuity of support [5], and psychological support [1]. Caregivers expect for high-quality, personalized assistance from healthcare professionals [1,15,37] and the need for allocation of time for themselves [24,26,42].

## 4. Discussion

This scoping review synthesizes data from 38 publications spanning a period of 34 years, the majority of which were published after 2010. Quantitative, qualitative, and mixed-method studies that used a variety of research tools were included. The review identified key domains related to the psychosocial well-being of informal caregivers of individuals receiving HMV. These domains included burden, mental health, quality of life, coping, perceived needs and sources of support, as well as the challenges of adapting to the caregiver role and the challenges of daily life but also positive aspects of caregiving. The results demonstrate the multidimensional nature of the caregiving experience and the commonality of many challenges faced by caregivers, regardless of the cultural context or healthcare system.

The review revealed that the majority of caregivers for individuals receiving home mechanical ventilation (HMV) are women, consistent with the existing literature on the global caregiving of individuals with chronic illnesses and disabilities [47]. This may influence both the nature of the burden they experienced and how they cope with it. These differences may be exacerbated by gender roles and cultural norms. Support should be gender sensitive and take social expectations into account [48].

Among the areas included in this review, the caregiver burden is one of the most extensively described. It refers to the subjective, multidimensional strain associated with long-term caregiving roles [49]. Both quantitative studies using standardized assessment tools and qualitative and mixed-methods research have demonstrated the caregiver burden might be mental, physical, emotional, or social [38]. Studies provide evidence that a significant burden on caregivers that affects their quality of life and mental health [13,14,17,18]. Notably, a study conducted in Iran reported that 91.3% of respondents experienced severe burden [14,17]. Gender differences have also been observed with women experiencing greater burden than men do [7,13,18]. This finding is consistent with the literature on caregiving in other populations, such as caregivers of older adults with chronic illness [50] and caregivers of stroke survivors [51]. These differences may have implications for targeted support interventions, which should be gender sensitive.

Findings regarding differences in burden levels between caregivers of individuals on IV and NIV were inconclusive; this may be due to the heterogeneity of the groups studied. While Karagün et al. [19] and Płaszewska et al. [7] reported higher burden levels among caregivers of IV individuals, studies by Kim & Kim [20] and Tülek et al. [13] revealed no differences between the two groups. These results may suggest that the type of ventilation is not the sole or dominant factor determining burden levels. In this context, it seems reasonable to consider implementing solutions such as respite care, which provides temporary relief for caregivers by enabling them to take short breaks from their caring responsibilities, although the evidence for its effectiveness is still inconclusive [52]. Additionally, care provided by long-term care facilities does not significantly differ in total caregiver strain compared with home care [41].

Anxiety and depression are among the most frequently reported mental health problems. Caregivers commonly described feeling overwhelmed, insecure, worrying about responsibility for the patient, and difficulty in predicting the patient’s condition [11,13,22,23,34,35]. In the Tülek et al. study [13], over half of the respondents manifested symptoms of anxiety (51.9%) and depression (57.7%), and these values correlated with the burden of caregiving. In the study by Lee et al. [34], as many as 64.4% had moderate symptoms of depression, and 23.7% had severe symptoms. High levels of burden are considered a significant risk factor for anxiety and depressive symptoms [53]. These data clearly indicate the urgent need for structured, system-level psychological support for informal caregivers.

Informal caregivers frequently encounter limitations in social interactions (including social isolation) and work activities, which can result in a decline in their quality of life [1,5,15,21,22,23,24,27,28,32]. In several Asian countries, the emphasis on family responsibilities and the social expectations of caregivers is emphasized, which can further increase pressure and guilt [29,34]. This is a particular concern because, as the literature indicates, elevated levels of loneliness may result in negative consequences for those receiving informal care [54].

Caregivers used various coping strategies, which included both adaptive and maladaptive approaches. Commonly reported strategies included reorientation of goals, planning, acceptance, and active coping [7,28], reflecting a constructive approach to overcoming caregiving difficulties and adapting to changing conditions. Smith et al. [32] noted that mobilizing social support is an important coping component. However, the use of maladaptive strategies, such as resignation, has also been observed in some cases [28].

Several studies have emphasized the role of spirituality as a source of psychological strength [18,23,29], which may indicate the importance of individual beliefs and internal resources in the adaptation process. Notably, it is not uncommon for caregivers to indicate a sense of meaning derived from being able to provide care in a home environment [1,30,45].

This review also highlights the variety of tools that were used to assess quality of life among caregivers of VAIs living at home, which complicates direct comparisons across studies. Baxter et al. [26] reported that caregivers exhibited lower quality of life compared to the general population, whereas Kim & Kim [20] and Tülek et al. [13] reported an association between quality of life and burden, which may suggest that a greater burden leads to a lower quality of life for caregivers. In contrast, the study by Kaub-Wittemer et al. [27] revealed no differences in quality of life between caregivers of IV- and NIV-ventilated individuals, which may suggest the influence of other more complex factors.

Caregivers face many challenges in their daily functioning. One of these limitations is the lack of adequate preparation for the caregiver role. Qualitative studies have highlighted inadequate training in caregiving knowledge and skills, the operation of medical equipment, and insufficient education on how to manage emergency situations [16,21,22,23,24,29,37]. Caregivers often have to learn through trial and error, which exacerbates their stress and feelings of uncertainty [5].

Although the initial intensity of training can be overwhelming, well-designed educational programs can effectively reduce caregiving anxiety [46]. The potential benefits of adequate preparation for the caregiver role have also been demonstrated among other populations [55].

In their daily lives, caregivers require high-quality, personalized, and continuous support from healthcare professionals [1,15,37] to reduce their feelings of insecurity [5]. Caregivers often expect help and appreciate the opportunity to share responsibilities [5,26]. However, ambiguity in assessing relationships with professionals suggests a tension between the need for help and the fear of losing autonomy and control over the home environment [43].

Moreover, some caregivers reported insufficient involved in therapeutic decision-making processes, which may negatively affect their perceived burden and satisfaction [5,16]. These findings underscore the importance of collaborative, family-centered care models that respect caregivers’ roles while providing appropriate professional guidance.

Additional burdens experienced by caregivers were identified in cross-country studies conducted during the COVID-19 pandemic. Caregivers reported increased anxiety, limited access to medical services, shortage in personal protective equipment, and increased social isolation [11]. The pandemic exposed systemic weaknesses in home care and highlighted the need for health systems to be better prepared for emergencies. Similar findings have emerged in studies among other caregiver populations [56].

Not all studies focused exclusively on the negative aspects of caregiving. Many caregivers experienced a sense of meaning, bonding with the care recipient and feeling fulfilled from being able to care for a loved one. Some also expressed that, given the choice, they would opt for respiratory support again [1,21,27,45]. A high quality of family relationship can serve as a protective factor, helping to mitigate the effects of caregiver burden. This finding is also supported by studies conducted in oncology populations [57].

### 4.1. Limitations

Despite the broad scope of the search, the use of selection procedures, and the exercise of due diligence, this scoping review is subject to several limitations. Although six databases were searched, it is possible that relevant publications may have been omitted, particularly those indexed in less accessible sources. Furthermore, restricting the review to English-language articles may have narrowed the perspective and affected the coverage of the analyzed issues.

Consistent with the nature of scoping reviews, no formal assessment of the methodological quality of the included studies was conducted, which increases the risk of including studies with varying levels of reliability. The variety of study types (quantitative, qualitative, and mixed), measurement tools, and cultural contexts may have complicated the synthesis and comparison of findings.

Moreover, inconsistencies in the definitions and operationalization of key concepts such as “burden”, “coping”, and “quality of life” across studies may have contributed to data ambiguity. Therefore, the results should be interpreted with caution, acknowledging these limitations.

### 4.2. Research Gaps and Future Directions

Despite the growing literature on caregivers of individuals receiving HMV, this review has revealed several significant research gaps.

A limited number of studies have employed a longitudinal design, making it difficult to evaluate changes in burden, quality of life, or mental health over time. Most studies are based on cross-sectional data, which precludes establishing causality and does not provide data on the dynamics of development.

Few studies have evaluated the effectiveness of interventions supporting caregivers, such as educational programs, respite care, or psychological support. Experimental and quasi-experimental studies are needed to assess the effectiveness and costs of such solutions.

The experiences of caregivers are deeply influenced by local cultural norms and healthcare systems. For instance, in many Asian countries, caregiving is strongly framed by familial obligations and loyalty toward ill family members [29,34]. In contrast, in countries such as the United States and Canada, greater emphasis is placed on autonomy, privacy, and institutional support [5,24].

This provides a basis for the development of more appropriate, contextually grounded interventions aimed at supporting both patients and their caregivers.

## 5. Conclusions

Caring for individuals receiving HMV is a complex, multifaceted experience that involves significant responsibility and mental strain, as well as emotional, social, and organizational challenges. This scoping review identified a wide range of difficulties faced by informal caregivers, including burden, mental health disorders, fatigue, deterioration in quality of life, information deficits, and limited access to supportive resources.

Constructive coping strategies, such as active coping, acceptance, and spirituality, which can serve a protective function, were also identified. Some caregivers also perceived their role as a source of satisfaction and meaning. Differences in caregiving experiences appear to be influenced primarily by three factors: the cultural context, the healthcare system, and individual resources.

This review highlights the need to implement multilevel support interventions that include both practical training and emotional, psychological, and organizational support. Such a comprehensive approach has the potential to reduce burden, improve the quality of life of caregivers, and strengthen their role within the care system for adults receiving HMV.

## Data Availability

All data extracted are presented in the article and Supplementary Materials; additional materials will be made available upon reasonable request.

## Supplementary Materials

The following supporting information can be downloaded at: https://www.mdpi.com/article/10.3390/jcm14176294/s1, Supplementary File S1: Scoping review protocol; Supplementary File S2: Search strategy; Supplementary File S3: PRISMA checklist.

## Abbreviations

The following abbreviations are used in this manuscript:

Ctrl.	Control group
HMV	Home mechanical ventilation
Inter.	Intervention group
IQR	Interquartile range
IV	Invasive ventilation
mo	Months
n	Sample size
n/a	Not applicable
NIV	Non-invasive ventilation
VAIs	Ventilator-assisted individuals
y	Years
