# Supplementary material for: Psychosocial Well-Being of Informal Caregivers of Adults Receiving Home Mechanical Ventilation: A Scoping Review
Source: J Clin Med. 2025 Sep 5;14(17):6294. doi: 10.3390/jcm14176294 (PMC12429581; doi:10.3390/jcm14176294)
Supplement: Supplementary file 1 [file jcm-14-06294-s001.zip › Supplementary File S1 Scoping review protocol-edited.pdf]

Psychosocial well-being of informal caregivers of adults receiving home mechanical ventilation: A scoping review protocol

Introduction

Home mechanical ventilation (HMV) improves the quality of life for patients with chronic respiratory failure; however, it significantly impacts informal caregivers who provide daily care. There is an insufficient number of comprehensive studies addressing the psychological and social dimensions of caregiving for ventilator-assisted individuals living at home. The planned scoping review will identify key aspects related to psychosocial well-being. Recognizing existing knowledge gaps will provide a foundation for further, more detailed research.

Review team

Jakub Cichoń[1]
Monika Homa[2]
Lucyna Płaszewska-Żywko[1]
Maria Kózka[1]
[1] Department of Specialized Nursing, Faculty of Health Sciences, Jagiellonian University – Medical College, Kraków, Poland
[2] Aestimo s.c., Kraków, Poland
Correspondence: Jakub Cichoń, jakub.cichon@uj.edu.pl

Timeline

November 2024 – June 2025

Aim

Explore and map the nature, extent, and scope of existing research on the psychosocial well-being of informal caregivers of adults receiving HMV.

Research questions

1. Which aspects of the psychosocial well-being of informal caregivers of adults receiving HMV are most frequently explored in the literature?
2. What research methods and instruments are employed to investigate the psychosocial well-being of these caregivers?
3. What knowledge gaps can be identified based on the literature?

Population, Concept, and Context (PCC) framework

- Population: informal, unpaid caregivers for adults receiving HMV (invasive or non-invasive);

- Concept: psychosocial well-being (e.g. burden, quality of life, needs, anxiety, depression, tiredness, support, and coping);
- Context: informal home-based care for patients using HMV.

Approach and methods

This review will be conducted and reported in accordance with the PRISMA extension for scoping reviews (PRISMA-ScR) guidelines.

The selection process will involve four steps:

1. Deduplication: All records retrieved from databases will be imported into Zotero for deduplication and management.
2. Title and abstract screening: Two independent reviewers (Jakub Cichoń and Monika Homa) will screen titles and abstracts against the eligibility criteria.
3. Full-text review: Full texts of potentially relevant studies will be independently assessed by the same reviewers.
4. Discrepancy resolution: Any disagreements will be discussed. If unresolved, Lucyna Płaszewska-Żywko will be consulted, and final decisions will be made by Maria Kózka.

The screening process will be documented and presented in a PRISMA flow diagram.

Data charting

Data will be extracted using a standardized charting form developed in Microsoft Excel. The form will include the following fields:

- Bibliographic details (authors, year, and journal);
- Study design;
- Study period;
- Demographic data (age, gender, and relationship to patient);
- Ventilator-assisted individuals' details (age, type of ventilation, and duration of HMV);
- Key concepts (e.g., burden, quality of life, anxiety, depression, coping, fatigue, social support, and needs);
- Measurement tools;
- Key findings.

Data analysis and synthesis

The extracted data will be summarized in tables and then analyzed using qualitative content analysis. The extracted data will be organized into key concepts and study characteristics relevant to the review's objectives.

Due to anticipated heterogeneity in study designs, populations, and outcomes, data will not be pooled. Instead, results will be reported narratively.

Information sources and search strategy

The review will identify quantitative, qualitative, and mixed studies published in peer-reviewed scientific journals. The following databases will be used:

- APA PsycInfo (via EBSCO);
- Embase;
- MEDLINE (via PubMed);
- Scopus;
- CINAHL Ultimate;
- Web of Science.

Search date: November 2024.
Time limits: no filters will be set.
Language limits: no filters will be set.

Search strategy:
Two domains (“caregivers” and “mechanical ventilation”) combined using “AND”. Within the domain, keywords are linked using “OR”.

Domain “caregivers”:

- Caregivers;
- Carers;
- Caregiving;
- Informal caregivers;
- Informal carers;
- Family caregivers;
- Family carers;
- Primary caregivers;
- Primary carers;
- Spouse caregivers;
- Spouse carers;
- Unpaid caregivers;
- Unpaid carers;
- Husband;
- Wife;
- Spouse;
- Dyads.

Domain “mechanical ventilation”:

- Home mechanical ventilation;
- HMV;
- Prolonged ventilation;

- Chronic ventilation;
- Long-term mechanical ventilation;
- Ventilator-dependent;
- Ventilator-assisted;
- Non-invasive mechanical ventilation;
- Noninvasive mechanical ventilation;
- Non-invasive ventilation;
- Noninvasive ventilation;
- Non-invasive face mask ventilation;
- Noninvasive face mask ventilation;
- Home non-invasive ventilation;
- Home noninvasive ventilation;
- Invasive mechanical ventilation;
- Invasive ventilation;
- Home invasive mechanical ventilation;
- Tracheostomy;
- Tracheotomy;
- Mechanical ventilation;
- Nocturnal ventilation;
- Chronic respiratory failure;
- Chronic ventilatory failure;
- Ventilator.

Eligibility criteria

Inclusion criteria:

- Primary, peer-reviewed, and original full-length research articles published in a scientific journal;
- Studies that report evidence on the psychosocial well-being of informal, unpaid caregivers for adults receiving HMV;
- Studies that employed quantitative, qualitative, or mixed-methods research methods;
- Full-text available online.

Exclusion criteria:

- Secondary research, nonoriginal publications, gray literature, or single case studies;
- Studies involving heterogeneous populations, in which data specific to informal caregivers of adults receiving HMV were not separately reported or extractable;
- Studies on caregivers of individuals using continuous positive airway pressure (CPAP) or home oxygen therapy only;
- Published in a language other than English.

Critical Appraisal

No formal quality assessment will be conducted, as per the scoping review methodology. However, methodological aspects such as the study design, sample size, and reliability of measures will be recorded during data extraction.

Registration

This protocol will not be registered.

Ethics

No ethical approval is required, as this review uses publicly available published data.
