# Supplementary material for: Psychosocial Well-Being of Informal Caregivers of Adults Receiving Home Mechanical Ventilation: A Scoping Review
Source: J Clin Med. 2025 Sep 5;14(17):6294. doi: 10.3390/jcm14176294 (PMC12429581; doi:10.3390/jcm14176294)
Supplement: Supplementary file 1 [file jcm-14-06294-s001.zip › Supplementary File S2 Search strategy-edited.pdf]

Search strategy

APA PsycInfo (via EBSCO)

(((home+mechanical+ventilation)+OR+(HMV)+OR+(prolonged+ventilation)+OR+(chronic+ventilation)+OR+(long-term+mechanical+ventilation)+OR+(ventilator-dependent)+OR+(ventilator-assisted)+OR+(non-invasive+mechanical+ventilation)+OR+(noninvasive+mechanical+ventilation)+OR+(non-invasive+ventilation)+OR+(noninvasive+ventilation)+OR+(non-invasive+face+mask+ventilation))+OR+((noninvasive+face+mask+ventilation)+OR+(home+non-invasive+ventilation)+OR+(home+noninvasive+ventilation)+OR+(invasive+mechanical+ventilation)+OR+(invasive+ventilation)+OR+(home+invasive+mechanical+ventilation)+OR+(tracheostomy)+OR+(tracheotomy)+OR+(mechanical+ventilation)+OR+(nocturnal+ventilation)+OR+(chronic+respiratory+failure)+OR+(chronic+ventilatory+failure))+OR+ventilator)+AND+((((caregivers)+OR+(carers)+OR+(caregiving)+OR+(informal+caregivers)+OR+(informal+carers)+OR+(family+caregivers)+OR+(family+carers)+OR+(primary+caregivers)+OR+(primary+carers)+OR+(spouse+caregivers)+OR+(spouse+carers)+OR+(unpaid+caregivers))+OR+((unpaid+carers)+OR+(husband)+OR+(wife)+OR+(spouse)+OR+(dyads)))+AND+(((home+mechanical+ventilation)+OR+(HMV)+OR+(prolonged+ventilation)+OR+(chronic+ventilation)+OR+(long-term+mechanical+ventilation)+OR+(ventilator-dependent)+OR+(ventilator-assisted)+OR+(non-invasive+mechanical+ventilation)+OR+(noninvasive+mechanical+ventilation)+OR+(non-invasive+ventilation)+OR+(noninvasive+ventilation)+OR+(non-invasive+face+mask+ventilation))+OR+((noninvasive+face+mask+ventilation)+OR+(home+non-invasive+ventilation)+OR+(home+noninvasive+ventilation)+OR+(invasive+mechanical+ventilation)+OR+(invasive+ventilation)+OR+(home+invasive+mechanical+ventilation)+OR+(tracheostomy)+OR+(tracheotomy)+OR+(mechanical+ventilation)+OR+(nocturnal+ventilation)+OR+(chronic+respiratory+failure)+OR+(chronic+ventilatory+failure))+OR+ventilator))

Embase

('caregivers'/exp OR caregivers OR 'carers'/exp OR carers OR 'caregiving'/exp OR caregiving OR 'informal caregivers' OR (informal AND ('caregivers'/exp OR caregivers)) OR 'informal carers' OR (informal AND ('carers'/exp OR carers)) OR 'family caregivers'/exp OR 'family caregivers' OR (('family'/exp OR family) AND ('caregivers'/exp OR caregivers)) OR 'family carers' OR (('family'/exp OR family) AND ('carers'/exp OR carers)) OR 'primary caregivers' OR (primary AND ('caregivers'/exp OR caregivers)) OR 'primary carers' OR (primary AND ('carers'/exp OR carers)) OR 'spouse caregivers' OR (('spouse'/exp OR spouse) AND ('caregivers'/exp OR caregivers)) OR 'spouse carers' OR (('spouse'/exp OR spouse) AND ('carers'/exp OR

carers)) OR 'unpaid caregivers' OR (unpaid AND ('caregivers'/exp OR caregivers)) OR 'unpaid carers' OR (unpaid AND ('carers'/exp OR carers)) OR 'husband'/exp OR husband OR 'wife'/exp OR wife OR 'spouse'/exp OR spouse OR dyads) AND ('home mechanical ventilation'/exp OR 'home mechanical ventilation' OR (('home'/exp OR home) AND mechanical AND ('ventilation'/exp OR ventilation)) OR hmv OR 'prolonged ventilation'/exp OR 'prolonged ventilation' OR (prolonged AND ('ventilation'/exp OR ventilation)) OR 'chronic ventilation' OR (chronic AND ('ventilation'/exp OR ventilation)) OR 'long-term mechanical ventilation' OR ('long term' AND mechanical AND ('ventilation'/exp OR ventilation)) OR 'ventilator dependent' OR 'ventilator assisted' OR 'non-invasive mechanical ventilation'/exp OR 'non-invasive mechanical ventilation' OR ('non invasive' AND mechanical AND ('ventilation'/exp OR ventilation)) OR 'noninvasive mechanical ventilation'/exp OR 'noninvasive mechanical ventilation' OR (noninvasive AND mechanical AND ('ventilation'/exp OR ventilation)) OR 'non-invasive ventilation'/exp OR 'non-invasive ventilation' OR ('non invasive' AND ('ventilation'/exp OR ventilation)) OR 'noninvasive ventilation'/exp OR 'noninvasive ventilation' OR (noninvasive AND ('ventilation'/exp OR ventilation)) OR 'non-invasive face mask ventilation' OR ('non invasive' AND ('face'/exp OR face) AND ('mask'/exp OR mask) AND ('ventilation'/exp OR ventilation)) OR 'noninvasive face mask ventilation' OR (noninvasive AND ('face'/exp OR face) AND ('mask'/exp OR mask) AND ('ventilation'/exp OR ventilation)) OR 'home non-invasive ventilation' OR (('home'/exp OR home) AND 'non invasive' AND ('ventilation'/exp OR ventilation)) OR 'home noninvasive ventilation' OR (('home'/exp OR home) AND noninvasive AND ('ventilation'/exp OR ventilation)) OR 'invasive mechanical ventilation'/exp OR 'invasive mechanical ventilation' OR (invasive AND mechanical AND ('ventilation'/exp OR ventilation)) OR 'invasive ventilation'/exp OR 'invasive ventilation' OR (invasive AND ('ventilation'/exp OR ventilation)) OR 'home invasive mechanical ventilation' OR (('home'/exp OR home) AND invasive AND mechanical AND ('ventilation'/exp OR ventilation)) OR 'tracheostomy'/exp OR tracheostomy OR 'tracheotomy'/exp OR tracheotomy OR 'mechanical ventilation'/exp OR 'mechanical ventilation' OR (mechanical AND ('ventilation'/exp OR ventilation)) OR 'nocturnal ventilation' OR (nocturnal AND ('ventilation'/exp OR ventilation)) OR 'chronic respiratory failure'/exp OR 'chronic respiratory failure' OR (chronic AND ('respiratory'/exp OR respiratory) AND ('failure'/exp OR failure)) OR 'chronic ventilatory failure'/exp OR 'chronic ventilatory failure' OR (chronic AND ventilatory AND ('failure'/exp OR failure)) OR 'ventilator'/exp OR ventilator)

MEDLINE (via PubMed)

((((((((((((((((caregivers) OR (carers)) OR (caregiving)) OR (informal caregivers)) OR (informal carers)) OR (family caregivers)) OR (family carers)) OR (primary caregivers)) OR (primary carers)) OR (spouse caregivers)) OR (spouse carers)) OR (unpaid caregivers)) OR (unpaid carers)) OR (husband)) OR (wife)) OR (spouse)) OR (dyads))

AND (((((((((((((((((((((((((home mechanical ventilation) OR (HMV)) OR (prolonged ventilation)) OR (chronic ventilation)) OR (long-term mechanical ventilation)) OR (ventilator-dependent)) OR (ventilator-assisted)) OR (non-invasive mechanical ventilation)) OR (noninvasive mechanical ventilation)) OR (non-invasive ventilation)) OR (noninvasive ventilation)) OR (non-invasive face mask ventilation)) OR (noninvasive face mask ventilation)) OR (home non-invasive ventilation)) OR (home noninvasive ventilation)) OR (invasive mechanical ventilation)) OR (invasive ventilation)) OR (home invasive mechanical ventilation)) OR (tracheostomy)) OR (tracheotomy)) OR (mechanical ventilation)) OR (nocturnal ventilation)) OR (chronic respiratory failure)) OR (chronic ventilatory failure)) OR (ventilator))

Scopus

Query 1:

(TITLE-ABS-KEY (caregivers) OR TITLE-ABS-KEY (carers) OR TITLE-ABS-KEY (caregiving) OR TITLE-ABS-KEY (informal AND caregivers) OR TITLE-ABS-KEY (informal AND carers) OR TITLE-ABS-KEY (family AND caregivers) OR TITLE-ABS-KEY (family AND carers) OR TITLE-ABS-KEY (primary AND caregivers) OR TITLE-ABS-KEY (primary AND carers) OR TITLE-ABS-KEY (spouse AND caregivers) OR TITLE-ABS-KEY (spouse AND carers) OR TITLE-ABS-KEY (unpaid AND caregivers) OR TITLE-ABS-KEY (unpaid AND carers) OR TITLE-ABS-KEY (husband) OR TITLE-ABS-KEY (wife) OR TITLE-ABS-KEY (spouse) OR TITLE-ABS-KEY (dyads))

Query 2:

(TITLE-ABS-KEY (home AND mechanical AND ventilation) OR TITLE-ABS-KEY (hmv) OR TITLE-ABS-KEY (prolonged AND ventilation) OR TITLE-ABS-KEY (chronic AND ventilation) OR TITLE-ABS-KEY (long-term AND mechanical AND ventilation) OR TITLE-ABS-KEY (ventilator-dependent) OR TITLE-ABS-KEY (ventilator-assisted) OR TITLE-ABS-KEY (non-invasive AND mechanical AND ventilation) OR TITLE-ABS-KEY (noninvasive AND mechanical AND ventilation) OR TITLE-ABS-KEY (non-invasive AND ventilation) OR TITLE-ABS-KEY (noninvasive AND ventilation) OR TITLE-ABS-KEY (non-invasive AND face AND mask AND ventilation) OR TITLE-ABS-KEY (noninvasive AND face AND mask AND ventilation) OR TITLE-ABS-KEY (home AND non-invasive AND ventilation) OR TITLE-ABS-KEY (home AND noninvasive AND ventilation) OR TITLE-ABS-KEY (invasive AND mechanical AND ventilation) OR TITLE-ABS-KEY (invasive AND ventilation) OR TITLE-ABS-KEY (home AND invasive AND mechanical AND ventilation) OR TITLE-ABS-KEY (tracheostomy) OR TITLE-ABS-KEY (tracheotomy) OR TITLE-ABS-KEY (mechanical AND ventilation) OR TITLE-ABS-KEY (nocturnal AND ventilation) OR TITLE-ABS-KEY (chronic AND respiratory AND failure) OR TITLE-ABS-KEY (chronic AND ventilatory AND failure) OR TITLE-ABS-KEY (ventilator))

Combining queries:
1 AND 2

CINAHL Ultimate
#45 #18 AND #44
#44 #19 OR #20 OR #21 OR #22 OR #23 OR #24 OR #25 OR #26 OR #27 OR #28 OR #29 OR #30 OR #31 OR #32 OR #33 OR #34 OR #35 OR #36 OR #37 OR #38 OR #39 OR #40 OR #41 OR #42 OR #43
#43 'ventilator'/exp OR ventilator
#42 'chronic ventilatory failure'/exp OR 'chronic ventilatory failure' OR ('chronic AND ventilatory AND ('failure'/exp OR failure))
#41 'chronic respiratory failure'/exp OR 'chronic respiratory failure' OR (chronic AND ('respiratory'/exp OR respiratory) AND ('failure'/exp OR failure))
#40 'nocturnal ventilation' OR (nocturnal AND ('ventilation'/exp OR ventilation))
#39 'mechanical ventilation'/exp OR 'mechanical ventilation' OR ('mechanical' AND ('ventilation'/exp OR ventilation))
#38 'tracheotomy'/exp OR tracheotomy
#37 'tracheostomy'/exp OR tracheostomy
#36 'home invasive mechanical ventilation' OR (('home'/exp OR home) AND invasive AND mechanical AND ('ventilation'/exp OR ventilation))
#35 'invasive ventilation'/exp OR 'invasive ventilation' OR (invasive AND ('ventilation'/exp OR ventilation))
#34 'invasive mechanical ventilation'/exp OR 'invasive mechanical ventilation' OR ('invasive AND mechanical AND ('ventilation'/exp OR ventilation))
#33 'home noninvasive ventilation' OR (('home'/exp OR home) AND noninvasive AND ('ventilation'/exp OR ventilation))
#32 'home non-invasive ventilation' OR (('home'/exp OR home) AND 'non invasive' AND ('ventilation'/exp OR ventilation)
#31 'noninvasive face mask ventilation' OR (noninvasive AND ('face'/exp OR face) AND ('mask'/exp OR mask) AND ('ventilation'/exp OR ventilation))
#30 'non-invasive face mask ventilation' OR ('non invasive' AND ('face'/exp OR face) AND ('mask'/exp OR mask) AND ('ventilation'/exp OR ventilation))

#29 'noninvasive ventilation'/exp OR 'noninvasive ventilation' OR (noninvasive AND ('ventilation'/exp OR ventilation))
#28 'non-invasive ventilation'/exp OR 'non-invasive ventilation' OR (non-invasive AND ('ventilation'/exp OR ventilation))
#27 'noninvasive mechanical ventilation'/exp OR 'noninvasive mechanical ventilation' OR ('noninvasive AND mechanical AND (ventilation'/exp OR ventilation))

#26 'non-invasive mechanical ventilation'/exp OR 'non-invasive mechanical ventilation' OR ('non invasive' AND mechanical AND ('ventilation'/exp OR ventilation))
#25 'ventilator assisted'
#24 'ventilator dependent'
#23 'long-term mechanical ventilation' OR ('long term' AND mechanical AND ('ventilation'/exp OR ventilation))
#22 'chronic ventilation' OR (chronic AND ('ventilation'/exp OR ventilation))
#21 'prolonged ventilation'/exp OR 'prolonged ventilation' OR ('prolonged' AND ('ventilation'/exp OR ventilation))
#20 hmv
#19 'home mechanical ventilation'/exp OR 'home mechanical ventilation' OR (('home'/exp OR home) AND mechanical AND ('ventilation'/exp OR ventilation)
#18 #1 OR #2 OR #3 OR #4 OR #5 OR #6 OR #7 OR #8 OR #9 OR #10 OR #11 OR #12 OR #13 OR #14 OR #15 OR #16 OR #17
#17 dyads
#16 'spouse'/exp OR spouse
#15 'wife'/exp OR wife
#14 'husband'/exp OR husband
#13 'unpaid carers' OR (unpaid AND ('carers'/exp OR carers))
#12 'unpaid caregivers' OR (unpaid AND ('caregivers'/exp OR caregivers))
#11 'spouse carers' OR (('spouse'/exp OR spouse) AND ('carers'/exp OR carers)
#10 'spouse caregivers' OR (('spouse'/exp OR spouse) AND ('caregivers'/exp OR caregivers))
#9 'primary carers' OR (primary AND ('carers'/exp OR carers))
#8 'primary caregivers' OR (primary AND ('caregivers'/exp OR caregivers))
#7 'family carers' OR (('family'/exp OR family) AND ('carers'/exp OR carers))
#6 'family caregivers'/exp OR 'family caregivers' OR (('family'/exp OR family) AND ('caregivers'/exp OR caregivers))
#5 'informal carers' OR (informal AND ('carers'/exp OR caregivers))
#4 'informal caregivers' OR (informal AND ('caregivers'/exp OR caregivers))
#3 'caregiving'/exp OR caregiving
#2 'carers'/exp OR carers
#1 'caregivers'/exp OR caregivers

Web of Science

Query #1

home mechanical ventilation (All Fields) or HMV (All Fields) or prolonged ventilation (All Fields) or chronic ventilation (All Fields) or long-term mechanical ventilation (All Fields) or ventilator-dependent (All Fields) or ventilator-assisted (All Fields) or non-invasive mechanical ventilation (All Fields) or noninvasive mechanical ventilation (All Fields) or non-invasive ventilation (All Fields) or noninvasive ventilation (All Fields)

or non-invasive face mask ventilation (All Fields) or noninvasive face mask ventilation (All Fields) or home non-invasive ventilation (All Fields) or home noninvasive ventilation (All Fields) or invasive mechanical ventilation (All Fields) or invasive ventilation (All Fields) or home invasive mechanical ventilation (All Fields) or tracheostomy (All Fields) or tracheotomy (All Fields) or mechanical ventilation (All Fields) or nocturnal ventilation (All Fields) or chronic respiratory failure (All Fields) or chronic ventilatory failure (All Fields) or ventilator (All Fields)

Query #2
caregivers (All Fields) or carers (All Fields) or caregiving (All Fields) or informal caregivers (All Fields) or informal carers (All Fields) or family caregivers (All Fields) or family carers (All Fields) or primary caregivers (All Fields) or primary carers (All Fields) or spouse caregivers (All Fields) or spouse carers (All Fields) or unpaid caregivers (All Fields) or unpaid carers (All Fields) or husband (All Fields) or wife (All Fields) or spouse (All Fields) or dyads (All Fields)

Combining queries:
#2 AND #1
